# Long‐Term Minocycline Treatment Exhibits Enhanced Therapeutic Effects on Ischemic Stroke by Suppressing Inflammatory Phenotype of Microglia Through the EMB/MCT4/STING Pathway

**DOI:** 10.1111/cns.70328

**Published:** 2025-03-26

**Authors:** Bo Cheng, Shangqi Liu, Ling Gao, Ning Xin, Zhenying Shang, Ziwen Zhu, Yang Yang, Rui Ma, Zixiang Xu, Jing Liu, Dunjing Wang

**Affiliations:** ^1^ Department of Neurology Tongji Hospital, School of Medicine, Tongji University Shanghai China; ^2^ Department of Psychiatry The Affiliated Xuzhou Eastern Hospital of Xuzhou Medical University Xuzhou China; ^3^ Department of Neurology The Affiliated Hospital of Xuzhou Medical University Xuzhou China; ^4^ Department of Neurology Xuzhou Central Hospital, Affiliated Xuzhou Clinical College of Xuzhou Medical University Xuzhou China; ^5^ Department of Pharmacy The Affiliated Huaihai Hospital of Xuzhou Medical University, the 71st Group Army Hospital of CPLA Army Xuzhou China; ^6^ Clinical College Xuzhou Medical Universtiy Xuzhou China; ^7^ Department of Neurology The Affiliated Xuzhou Municipal Hospital of Xuzhou Medical University, Xuzhou No. 1 People's Hospital Xuzhou China

**Keywords:** glycolysis, ischemic stroke, microglia, minocycline, neuroinflammation

## Abstract

**Background:**

Neuroinflammation caused by excessive activation of microglia is a significant cause of poor prognosis in ischemic stroke patients. Minocycline, a microglial cell inhibitor, has neuroprotective effects in stroke, but its optimal treatment duration and specific mechanisms of action remain unclear. This study aimed to compare the efficacy of different minocycline treatment durations on stroke and explore their mechanisms of action.

**Methods:**

We investigated the effects of various durations of minocycline treatment on microglial polarization using cellular and animal models. The mechanisms of long‐term minocycline therapy for neuroprotective effects were explored through in vitro and in vivo experiments.

**Results:**

In stroke models, long‐term minocycline treatment showed a stronger inhibitory effect on neuroinflammation and improved neuron viability compared with short‐term treatment. Further in vitro and in vivo results indicated that long‐term minocycline treatment downregulated microglial glycolysis levels through the EMB/MCT4 axis, promoting the transformation of microglia to an anti‐inflammatory phenotype by inhibiting the activation of the STING pathway, thereby improving post‐stroke neuroinflammation.

**Conclusion:**

Long‐term minocycline therapy exerts neuroprotective effects in ischemic stroke by regulating the EMB/MCT4/STING axis and inhibiting the inflammatory phenotype of microglia through downregulating cellular glycolysis levels. Extending the treatment duration of minocycline appropriately may further improve ischemic stroke outcomes.

## Graphical abstract

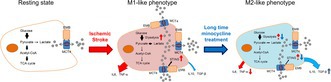

## 1 Introduction

Stroke is the second leading cause of adult mortality worldwide, with ischemic stroke accounting for approximately 84% of all strokes [1]. Excessive activation of microglia after stroke exacerbates neuroinflammatory damage [1, 2]. Current treatments for ischemic stroke mainly rely on thrombolysis with drugs such as rt‐PA and physical thrombectomy [3, 4], with limited options to alleviate poststroke neuroinflammatory damage, significantly affecting patient prognosis [5].

Minocycline, a tetracycline antibiotic, has been found to inhibit microglial activation and promote the transformation of microglia into anti‐inflammatory phenotypes, providing neuroprotective effects in ischemic stroke [6, 7, 8, 9, 10, 11]. Despite its efficacy in animal models, some clinical trials have not shown significant therapeutic effects [12, 13, 14, 15, 16]. For instance, a study by Kohler et al. reported that five consecutive intravenous injections of 100 mg minocycline did not significantly improve patient prognosis [17]. Another study found no significant therapeutic effects with continuous use of minocycline for 10 days in ischemic stroke patients [12]. Research suggests that the level of Inflammatory microglia continues to increase within 2 weeks of ischemic injury, while current clinical studies mainly focus on medication duration of 3–5 days, potentially explaining the inconsistent therapeutic effects observed in clinical trials compared with animal experiments [18, 19, 20, 21]. Given the neuroprotective effect of inhibiting excessive microglial activation in stroke, we hypothesize that longer minocycline therapy duration may enhance its neuroprotective effects compared with shorter duration [19, 22, 23, 24].

The mechanism by which minocycline inhibits microglial activation remains unclear, with changes in glycolytic metabolism levels being a potential pathway [25, 26]. After stroke, microglial activation shifts from oxidative phosphorylation to aerobic glycolysis, promoting activation and transformation to a proinflammatory phenotype while inhibiting phagocytosis of nerve‐damaging factors, exacerbating neuroinflammation [27, 28, 29, 30, 31]. Inhibiting microglial glycolytic metabolism may be an effective treatment for reducing neuroinflammation.

Embigin (EMB), a member of the immunoglobulin superfamily, is highly expressed in microglia and regulates cellular glycolytic levels in various diseases and tumors [32, 33]. However, its role in microglial glycolytic metabolism in stroke and its involvement in minocycline‐induced inhibition of microglial activation have not been explored. The monocarboxylate transporter (MCT) family is important lactate transporters that exert lactate transport function by binding to EMB or basigin [34, 35, 36]. MCT4 is the most important MCT family member in microglia involved in various neural functions such as synaptic pruning [37]. The stimulator of interferon genes (STING) pathway, involved in DNA‐mediated innate immune responses, also affects neuroinflammation in stroke and microglial inflammatory transformation, with studies showing that it upregulates cellular glycolytic metabolism in various diseases [38, 39, 40, 41, 42, 43, 44]. Therefore, it is of great significance to clarify whether minocycline exerts its therapeutic effect through the EMB/MCT4/STING axis.

In this study, we established a microglial oxygen–glucose deprivation (OGD) model and an animal middle cerebral artery occlusion (MCAO) model to compare the neuroprotective effects of minocycline at different durations and explore its specific mechanisms of action.

## 2 Materials and Methods

### 2.1 Cell Culture

HMC3 cells were maintained in minimum essential medium (MEM) (Cell research, China) with 10% fetal bovine serum (FBS) (Gibco, USA) and 1% penicillin–streptomycin (Beyotime, China) at 37°C in a 5% CO_2_ atmosphere. BV2 cells were cultured in high‐glucose Dulbecco's modified Eagle's medium (DMEM) (Gibco, USA) supplemented with 10% FBS (Gibco, USA), 1% penicillin–streptomycin (Beyotime, China), and 1% glutamine (Beyotime, China) under identical conditions. SH‐SY5Y cells were maintained in high glucose DMEM with 10% FBS and 1% penicillin–streptomycin at 37°C in a 5% CO_2_ atmosphere.

For OGD treatment, HMC3, BV2, and SH‐SY5Y cells were exposed to a hypoxic environment (0.2% O_2_, 94.8% N_2_ and 5% CO_2_) in serum/glucose‐free culture medium for 4 h at 37°C. The medium was then changed to complete medium, and the plates were transferred to normal conditions (95% air and 5% CO_2_).

### 2.2 Drug Treatment and Lentivirus Transfect

For minocycline treatment after OGD, cells were first cultured in complete medium with or without 50 μM minocycline (MCE, China) for 6 h; then, the medium was changed, and cells were further cultured for 12 h. For the control group, cells were cultured in complete medium without minocycline for the first 6 h and the following 12 h. For the short‐term treatment group, cells were first cultured in complete medium with minocycline for 6 h, then changed to complete medium without minocycline for 12 h. For the long‐term treatment group, cells were always cultured in complete medium with minocycline.

For drug combination treatment, the concentration of minocycline was 50 μM, and the concentrations of VB124 (MCE, China) and SN‐011 (MCE, China) were 10 μM and 1 μM, respectively [45, 46].

Lentiviral transfection was performed when the cell density in the 24‐well plate reached 30%–50% confluence. After removing the original complete culture medium, 500 μL of complete medium containing lentivirus (Genechem, China) was added to each well for transfection. Following 24 h of incubation, the medium containing the lentivirus was replaced with fresh complete medium. The cells were then maintained in culture and subjected to puromycin selection to establish stable transfected cell lines.

### 2.3 Protein Extraction and Western Blot

Cells were treated at 4°C for 30 min with 1 mL of lysis buffer containing protease and phosphatase inhibitors (Beyotime, China). The supernatant was collected by centrifugation at 12,000 *g* for 30 min. Proteins were separated by gel electrophoresis, transferred to a membrane, blocked with a blocking reagent (Beyotime, China) for 15 min, washed three times, and incubated overnight with the primary antibody (Abcam, USA). The membrane was washed and incubated with a fluorescently tagged secondary antibody (Abcam, USA) for 2 h. After a final wash, the membrane was scanned to determine band intensities.

### 2.4 mRNA Extraction and qRT‐PCR

Total RNA was extracted using the TRIzol (Invitrogen, USA) method, and RNA was reverse transcribed into cDNA with a Vic qRT Super Kit (Vicmed, China) in 20 μL reactions. Real‐time quantitative PCR was performed using a LightCycler 480 (Roche, USA), with GAPDH as the internal reference. The target gene expression levels were calculated using the 2^−ΔΔCt^ method. Primer sequences were as follows:

hEMB forward: 5′‐TATTCCAATTAGGCGAGAGTGAAGAAC.

hEMB reverse: 5′‐AAGAATCACCTCAGCCACTATTACAAG.

mEMB forward: 5′‐TTGAACTTGATGAATGTGACTTGGAAG.

mEMB reverse: 5′‐CCTGTACTGACTGGTTAAGGTGTTG.

hMCT4 forward: 5′‐CGCCTTCCTGCTCACCATCC.

hMCT4 reverse: 5′‐GCTGAAGAGGTAGACGGAGTAGG.

mMCT4 forward: 5′‐CTCCACAGCCACTGACTACGG.

mMCT4 reverse: 5′‐GCAGCAGCACGAGACCAATG.

hGAPDH forward: 5′‐TGACTTCAACAGCGACACCCA.

hGAPDH reverse: 5′‐CACCCTGTTGCTGTAGCCAAA.

mGAPDH forward: 5′‐GGCAAATTCAACGGCACAGTCAAG.

mGAPDH reverse: 5′‐TCGCTCCTGGAAGATGGTGATGG.

### 2.5 Cell Viability Test

Cells were seeded into 96‐well plates at a density of 1 × 10^4^ cells per well overnight. After treatment, 20 μL of CCK8 (Vicmed, China) reagent was added to each well, the plate was incubated in the dark for 30 min, and absorbance at 450 nm was detected using a microplate reader.

### 2.6 Quantification of Glycolysis and Lactate Levels

Glucose uptake and lactate levels were quantified using glucose uptake assay kits (Dojindo, Japan) and lactate assay kits (Jiancheng, China) following the manufacturer's instructions. Relative glucose uptake and lactate levels were determined based on standard curves derived from standard samples.

### 2.7 Enzyme‐Linked Immunosorbent Assay

The supernatant of HMC3 and BV2 cells was collected by centrifugation at 500 g for 5 min at 4°C. The levels of interleukin 6 (IL‐6), interleukin 10 (IL‐10), tumor necrosis factor‐α (TNF‐α), and transforming growth factor‐β (TGF‐β) were measured using specific ELISA kits (Jianglai, China) according to the manufacturer's instructions.

### 2.8 Cell Immunofluorescence Staining

Cells were fixed with 4% paraformaldehyde (Beyotime, China) at room temperature for 30 min, washed with PBS three times, permeabilized and blocked with 0.1% TX‐100 (Vicmed, China) and 1% BSA (Vicmed, China) for 30 min, and incubated with the primary antibody (Abcam, USA) (1:2000) at 4°C overnight. Then, the cells were washed and incubated with a fluorescent secondary antibody (Abcam, USA) (1:500) for 1 h. The nucleus was stained with DAPI (Beyotime, China) for 15 min, and the cells were visualized using a fluorescence microscope.

### 2.9 Flow Cytometry

Digested and centrifuged the processed test cells, washed the cells three times with PBS to remove the culture medium. Resuspended the cells with PBS and added FITC Annexin staining solution (Keygen, China) to the test cells. Mixed the staining solution with the cells and incubated the cell suspension at room temperature in the dark for 15 min. Added an appropriate amount of PI staining solution (Keygen, China) to the cell suspension, mixed well again, and incubated at room temperature for 15 min. After incubation, detected by flow cytometer.

### 2.10 Animals

Female SD rats (Jihui, China) (180–200 g) were purchased and acclimated for 7 days before the experimental operation. Rats were kept in an environment maintained at 20°C ± 2°C, 55% humidity, and subjected to a 12‐h light/12‐h dark cycle with free access to food and water.

### 2.11 Animal Model Induction and Treatment

For the MCAO/R rat model, the right common carotid artery, external carotid artery, and internal carotid artery were exposed after the rats were anesthetized with isoflurane (RWD, China). A V‐shaped oblique incision was made on the right external carotid artery wall, a nylon wire was inserted into the ICA through the common carotid artery, and removed after 2 h to restore perfusion. Sham surgery rats underwent similar procedures without occluding the middle cerebral artery.

The animal model received intraperitoneal injections of 50 mg/kg/d minocycline or control solvent. The specific processing process is shown in Figure 7A.

### 2.12 Behavioral Tests

#### 2.12.1 Longa Score

Neurological deficits were assessed using the Longa score, a 5‐point scale where 0 indicates no deficits and 5 indicates death: 0—normal, no neurological deficits; 1—mild deficit, inability to fully extend the left forepaw; 2—moderate deficit, circling to the left; 3—severe deficits, falling to the left; 4—no spontaneous movement, decreased consciousness; 5—death [47].

#### 2.12.2 Open Field Test

Rats were placed in a 100 cm × 40 cm opaque methacrylate box, and their movements were recorded for 5 min. The total distance traveled and average speed were analyzed [48].

#### 2.12.3 Balance Beam Test

Rats were placed at one end of a 150 cm × 2.5 cm beam, and their ability to traverse the beam was observed. Performance was scored from 0 to 5, with higher scores indicating poorer neurological and behavioral recovery [49].

#### 2.12.4 Rotating Rod Test

Rats were placed on a rotating rod that gradually increased in speed from 4 rpm to 40 rpm over 5 min. The time taken for the rat to fall off was recorded [50].

#### 2.12.5 Forced Swimming Test

Rats were allowed to swim in a transparent container filled with 25°C water for 5 min. The total time the rats remained immobile was recorded, and the proportion of immobility time was calculated [48].

#### 2.12.6 Sucrose Preference Test

Before the test, rats were deprived of food and water for 24 h. During the test, they had free access to water and 1% sucrose solution for 1 h. The sucrose preference index was calculated as: sucrose preference = sucrose solution intake (g)/(sucrose solution intake (g) + water intake (g)) [51].

### 2.13 Tissue Staining

TTC staining: To evaluate cerebral infarct volume, brain tissue was quickly removed after euthanasia and frozen at −20°C for 30 min. The brain was then sliced and stained with 2% TTC dye (Servicebio, China) in the dark for 30 min at 37°C, with gentle shaking to ensure even staining. After staining, the slices were washed with PBS and observed.

#### 2.13.1 Immunofluorescence Staining

To assess microglial polarization, brain tissue was collected and fixed with paraformaldehyde. The tissue was then embedded in paraffin to create wax blocks. After slicing, dewaxing, and dehydration, the primary antibody was incubated overnight at 4°C. Subsequently, the secondary antibody was incubated at room temperature for 1 h after the primary antibody was discarded and rinsed with PBS. Finally, DAPI was used to stain the nuclei, and antifluorescence quenching agents (Beyotime, China) were applied to seal the sections before observation.

#### 2.13.2 HE Staining

HE staining was performed on the main organs of the animals to observe histological changes. The main organs were collected, fixed with 4% paraformaldehyde, embedded in paraffin, and sectioned. After dewaxing and hydration, HE staining was carried out and observed [7].

### 2.14 Patient Data and Bioinformatics Analyses

This study utilized data from the Human Protein Atlas database (https://www.proteinatlas.org/ENSG00000170571‐EMB/brain; https://www.proteinatlas.org/ENSG00000170571‐EMB/single+cell+type/brain) and the Gene Expression Omnibus (GEO) database Series GSE202518 (https://www.ncbi.nlm.nih.gov/geo/) [52, 53, 54, 55, 56].

### 2.15 Statistical Analysis

Data were obtained from at least three independent experiments unless otherwise specified, analyzed using SPSS 22.0 or GraphPad Prism 8.0, and expressed as mean ± standard deviation. Student's *t*‐tests and one‐way ANOVA were used to assess differences between groups. Kaplan–Meier survival analysis estimated survival distributions, with a two‐sided *p* value < 0.05 indicating statistical significance.

## 3 Results

### 3.1 Long‐Term Minocycline Treatment Further Inhibits Microglial Inflammatory Phenotype and Glycolysis Levels Compared to Short‐Term Treatment

**FIGURE 1:**
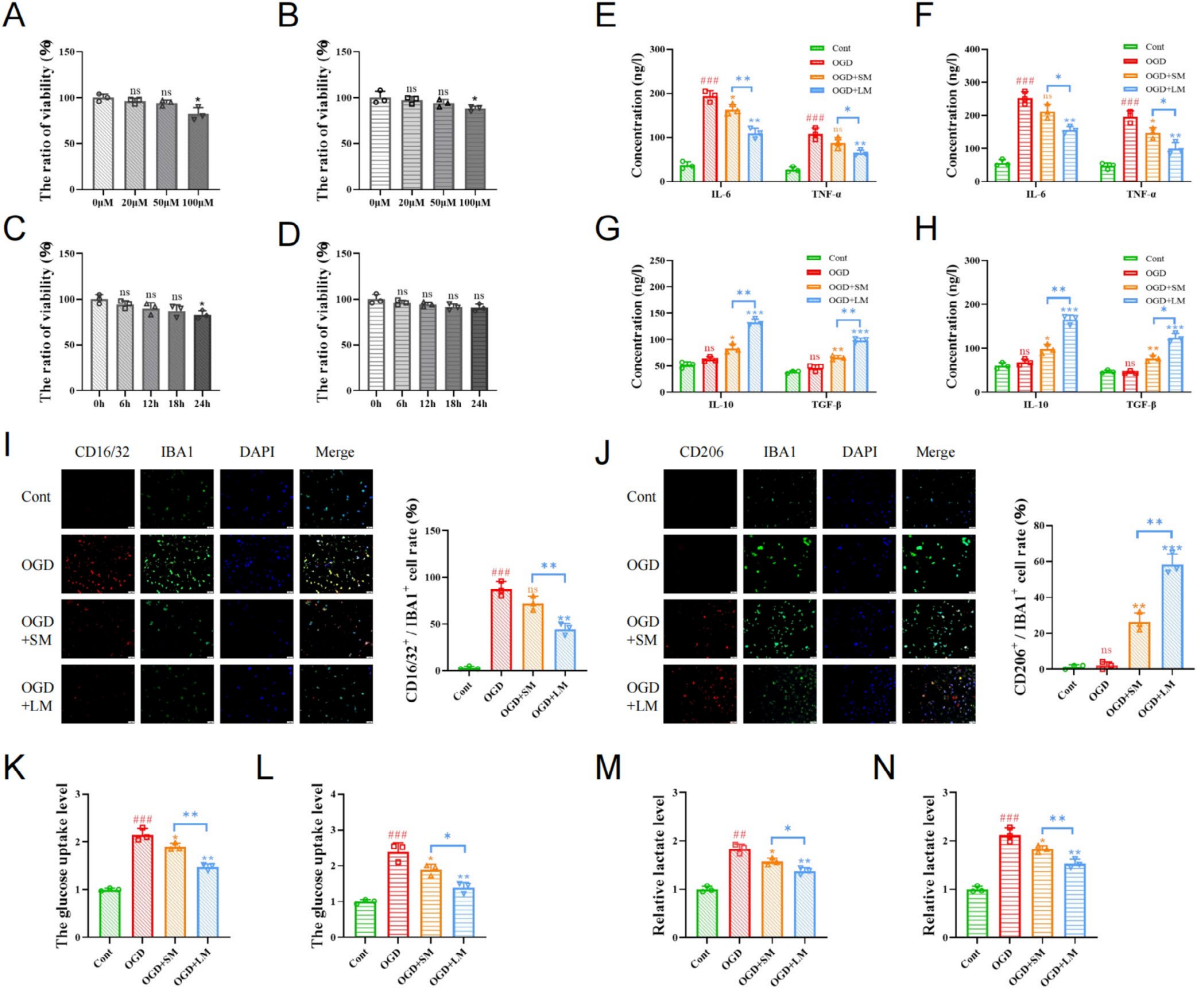
Long‐term minocycline treatment further reduces inflammation levels in post‐OGD microglia. (A, B) The viability of HMC3 cells (A) and BV2 cells (B) at different minocycline concentrations measured by CCK8 assays. (C, D) The viability of HMC3 cells (C) and BV2 cells (D) treated with 50 μM minocycline for various durations measured by CCK8 assays. (E, F) The levels of IL‐6 and TNF‐α in the supernatants of HMC3 cells (E) and BV2 cells (F) cultures measured by ELISA assay. (G, H) The levels of IL‐10 and TGF‐β in the supernatants of HMC3 cells (G) and BV2 cells (H) cultures measured by ELISA assay. (I, J) Immunofluorescence showing the proportion of double‐positive cells in HMC3 cells (I) and BV2 cells (J). Scale bar = 50 μm. (K, L) The glucose uptake level of HMC3 cells (K) and BV2 cells (L). (M, N) The lactate level of HMC3 cells (M) and BV2 cells (N). # indicates comparison between Cont and OGD groups, ns *p* > 0.05; ##*p* < 0.01; ###*p* < 0.001; *p < 0.05; **p < 0.01; ***p < 0.001.

We first determined the appropriate concentration of minocycline for this study through CCK8 assays, identifying 50 μM as the optimal concentration. Treating cells with 50 μM minocycline for 6 h, 12 h, and 18 h did not significantly affect cell viability. Therefore, we chose 6 h and 18 h as the short‐term and long‐term treatment durations, respectively (Figure 1A–D). ELISA results indicated that minocycline treatment downregulated IL‐6 and TNF‐α levels in post‐OGD HMC3 and BV2 cells, while upregulating IL‐10 and TGF‐β levels. Moreover, the changes in inflammatory factors were more pronounced in the long‐term treatment group compared with the short‐term group (Figure 1E–H). Immunofluorescence results showed that both 6‐h and 18‐h minocycline treatments reduced CD16/32 levels and increased CD206 levels in OGD‐treated cells, with the 18‐h treatment showing more significant effects (Figure 1I,J).

Following OGD treatment, we observed a significant increase in glycolysis levels in microglia (Figure 1K–N). Data from the HPA database showed that EMB is widely expressed in the human brain, with higher expression levels in microglia compared with other glial cells (Figure S1A,B). Analysis of the GEO database revealed higher EMB levels in the peripheral blood of patients with severe stroke compared to those with moderate stroke, suggesting a correlation between EMB and symptom severity (Figure S1C).

### 3.2 Minocycline Downregulates Microglial Glycolysis Levels by Inhibiting EMB Expression

**FIGURE 2:**
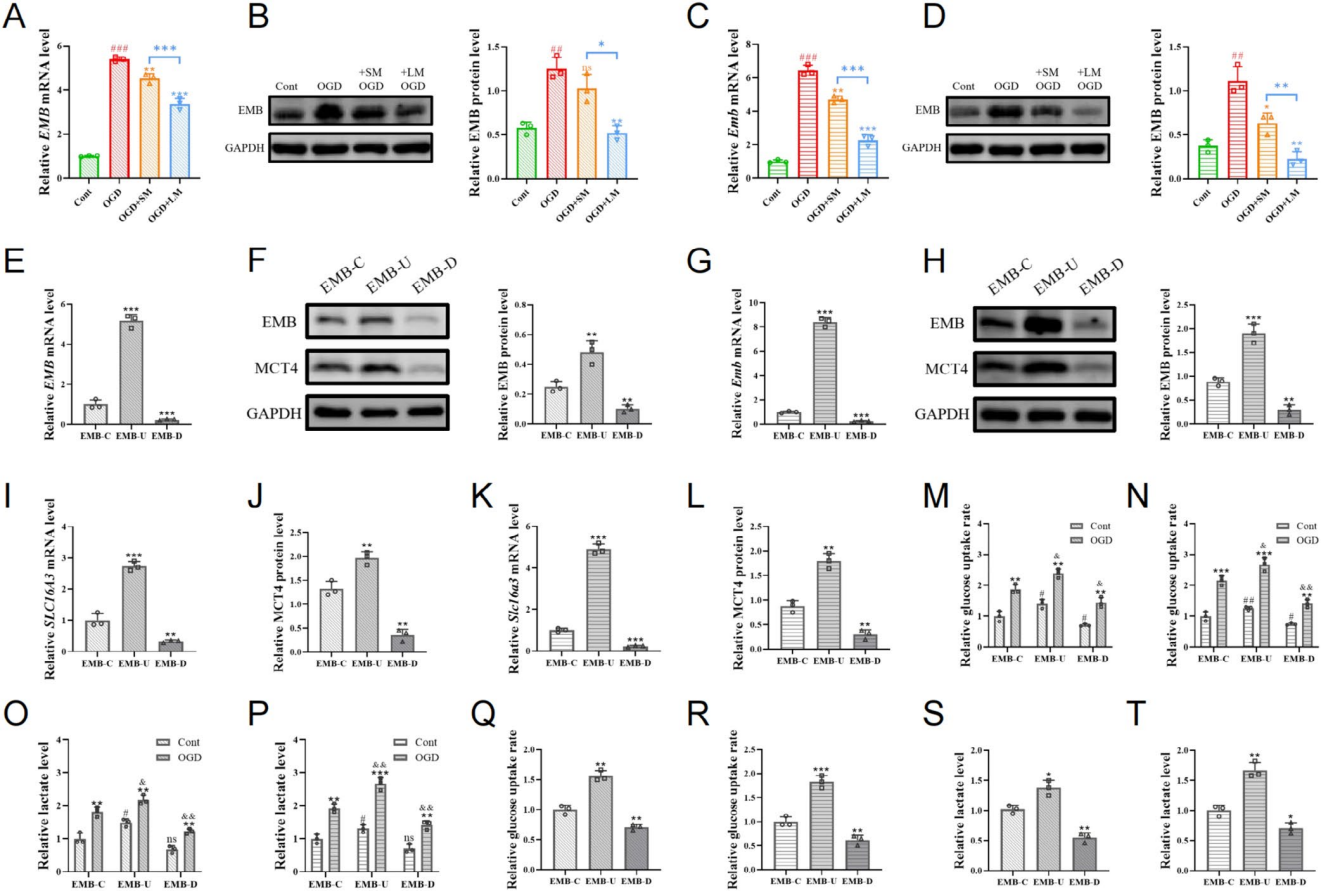
EMB regulates glycolytic metabolism in post‐OGD microglia. (A‐D) MRNA and protein levels of EMB in HMC3 cells (A, B) and BV2 cells (C, D). #indicates comparison between Cont and OGD groups, ##*p* < 0.01, ###*p* < 0.001. (E–H) MRNA and protein levels of EMB in virus‐transfected HMC3 cells (E, F) and BV2 cells (G, H). (I–L) MRNA levels of SLC16A3 and protein levels of MCT4 in virus‐transfected HMC3 cells (I, J) and BV2 cells (K, L). M‐P: Glucose uptake levels and lactate levels in virus‐transfected HMC3 cells (M, O) and BV2 cells (N, P). # indicates comparison under control conditions between EMB‐U, EMB‐D, and EMB‐C groups; EMB‐C, Cells transfected with control virus; EMB‐D, Cells transfected with EMB downregulated virus; EMB‐U, Cells transfected with EMB upregulated virus. ns *p* > 0.05; #*p* < 0.05; ##*p* < 0.01; & indicates comparison under OGD conditions between EMB‐C, EMB‐U, and EMB‐D groups, &*p* < 0.05, &&*p* < 0.01. Q‐T: Glucose uptake levels and lactate levels in post‐OGD HMC3 cells (Q, S) and post‐OGD BV2 cells (R, T) after long‐term minocycline treatment. *p < 0.05; **p < 0.01; ***p < 0.001.

PCR and Western blot analyses demonstrated that OGD treatment upregulated EMB mRNA and protein levels in HMC3 and BV2 cells, which were subsequently inhibited by minocycline in a time‐dependent manner (Figure 2A–D). We then established and verified EMB overexpression and knockdown cell lines using lentivirus (Figure 2E–H). Assays showed that overexpression of EMB increased microglial glycolysis, while knockdown of EMB reduced it (Figure 2I–L). OGD treatment increased glycolysis in all cell groups, with higher glycolysis levels in EMB‐overexpressing cells and lower levels in EMB knockdown cells compared to controls (Figure 2M–P). Long‐term minocycline treatment of OGD‐treated cells transfected with EMB virus revealed that glucose uptake and lactate levels remained higher in EMB‐overexpressing cells, whereas glycolysis levels further decreased in EMB knockdown cells (Figure 2Q–T).

### 3.3 Stroke Upregulates Inflammatory Levels of Microglia Through the EMB/MCT4 Axis

**FIGURE 3:**
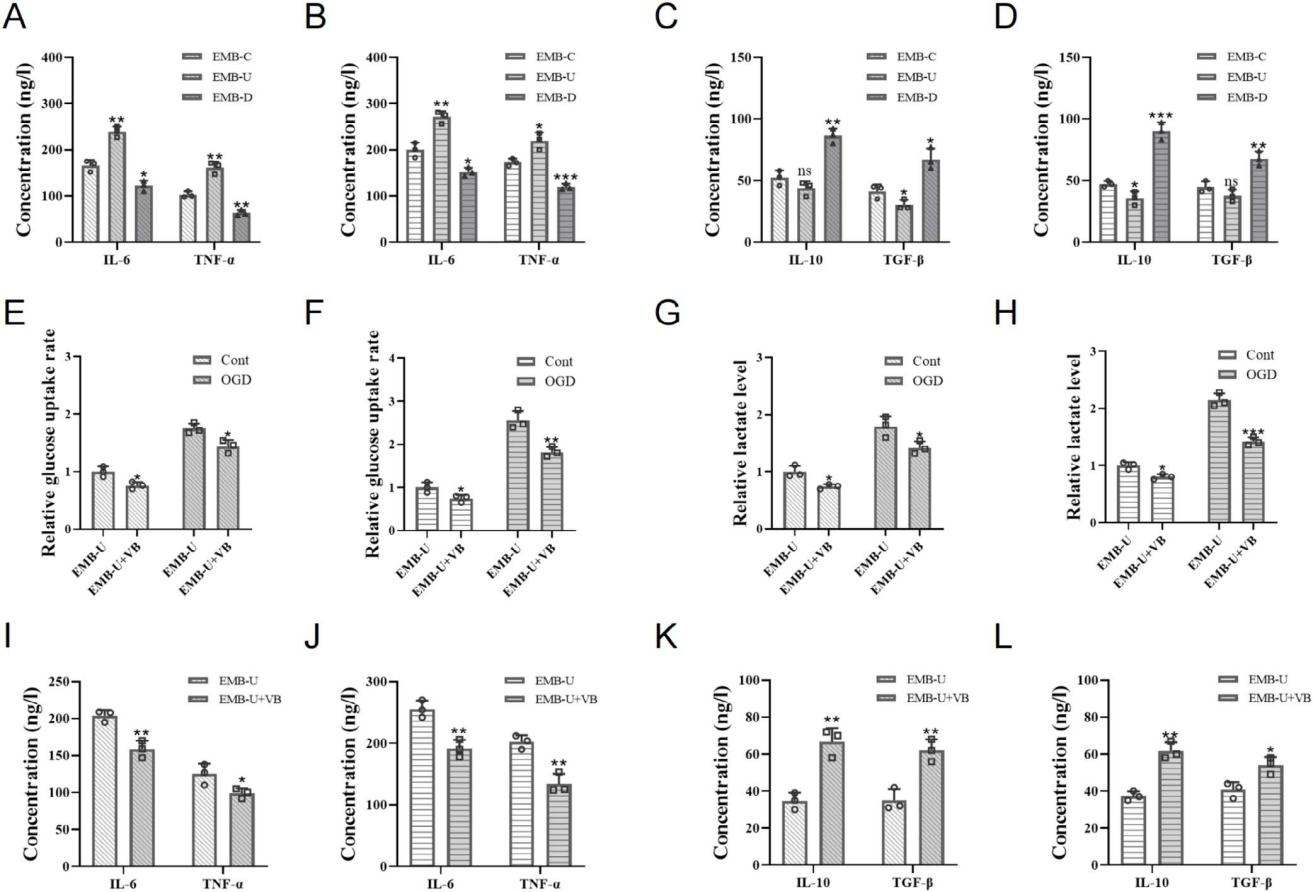
EMB upregulates inflammation levels and glycolytic metabolism in post‐OGD microglia via MCT4. (A–D) The levels of IL‐6, TNF‐α, IL‐10, and TGF‐β in virus‐transfected post‐OGD HMC3 cells (A, C) and BV2 cells (B, D). (E–H) Glucose uptake levels and lactate levels in EMB‐overexpressing HMC3 cells (E, G) and BV2 cells (F, H) treated with MCT4 inhibitor VB124. I‐L: The levels of IL‐6, TNF‐α, IL‐10, and TGF‐β in EMB‐overexpressing HMC3 cells (I, K) and BV2 cells (J, L) treated with MCT4 inhibitor VB124. ns *p* > 0.05; *p < 0.05; **p < 0.01; ***p < 0.001.

To investigate the role of EMB in stroke‐induced microglial inflammatory transformation, microglia modified with the EMB gene were exposed to OGD, and the level of IL‐6 and TNF‐α was higher in the supernatant of microglia overexpressing EMB, while a lower level of IL‐10 and TGF‐β compared to EMB knockdown microglia (Figure 3A–D). MCT4, an important lactate transporter and companion factor of the EMB family, mediates the cellular glycolytic response. EMB regulates MCT4's mRNA and protein levels in microglia (Figure 2C–F). Inhibiting MCT4 downregulated glycolysis in OGD‐treated EMB‐overexpressing cells (Figure 3E–H) and significantly inhibited their inflammatory levels (Figure 3I–L).

**FIGURE 4:**
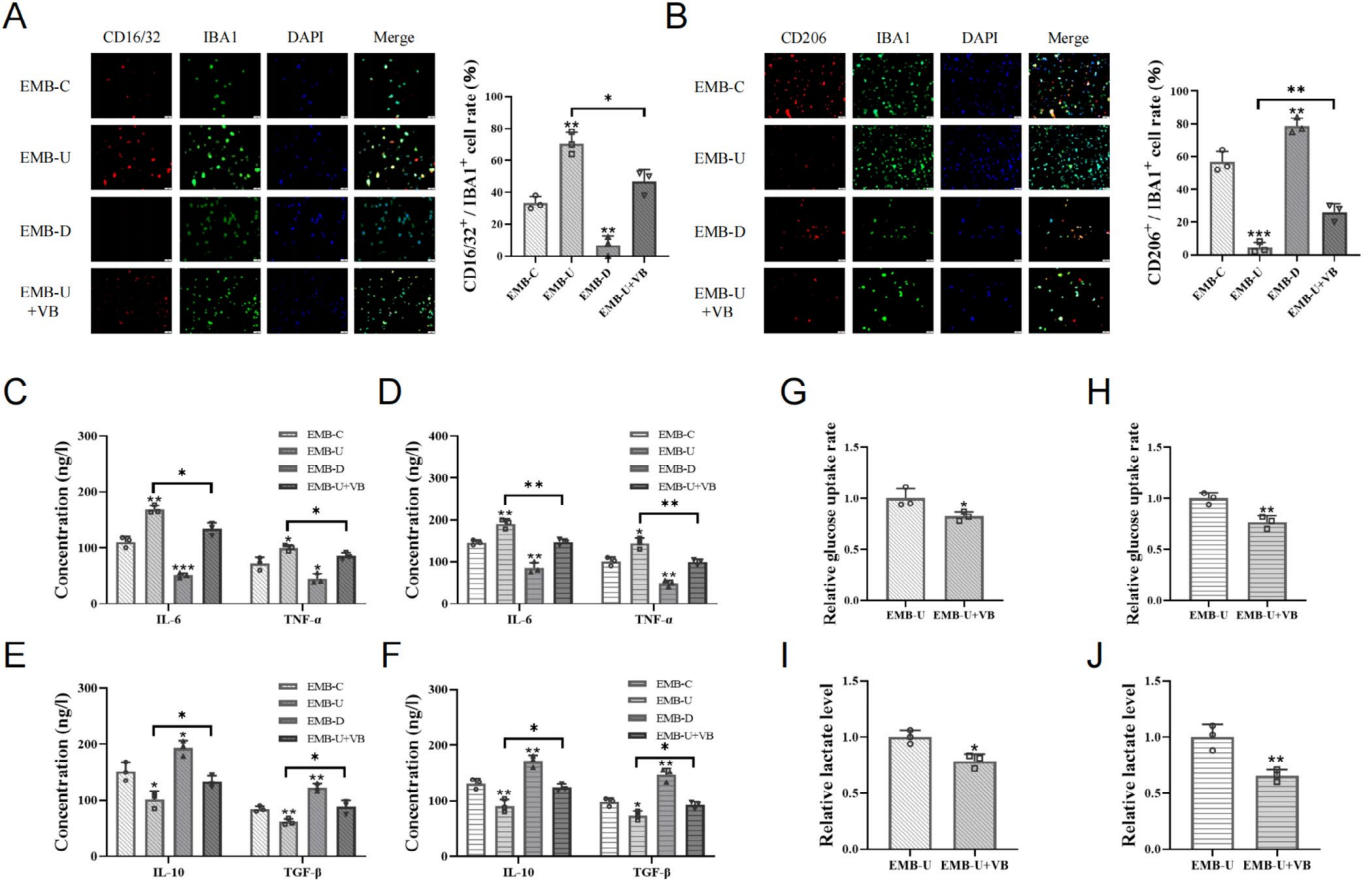
Microglial EMB levels influenced the anti‐inflammatory effects of long‐term minocycline. (A, B) Immunofluorescence showing the proportion of double‐positive cells in HMC3 cells (A) and BV2 cells (B) treated with long‐term minocycline post‐OGD. Scale bar = 50 μm. (C–F) The levels of IL‐6, TNF‐α, IL‐10, and TGF‐β in post‐OGD HMC3 cells (C, E) and BV2 cells (D, F) treated with long‐term minocycline. (G–J) Glucose uptake levels and lactate levels in post‐OGD EMB‐overexpressing HMC3 cells (G, I) and BV2 cells (H, J) treated with long‐term minocycline and VB124. *p < 0.05; **p < 0.01; ***p < 0.001.

After OGD and long‐term minocycline treatment, microglia overexpressing EMB upregulated CD16/32 while decreasing CD206 compared to EMB knockdown cells (Figure 4A,B). ELISA results showed that EMB overexpression promoted the release of more proinflammatory factors IL‐6 and TNF‐α, whereas EMB inhibition upregulated IL‐10 and TGF‐β levels in the cell supernatant (Figure 4C–F). Additionally, inhibiting MCT4 in EMB‐overexpressing cells reversed EMB‐induced glycolysis upregulation (Figure 4G–J) and inhibited the transformation to the inflammatory phenotype (Figure 4A,B).

### 3.4 EMB Counteracts the Anti‐Inflammatory Effect of Minocycline by Activating the STING Pathway

**FIGURE 5:**
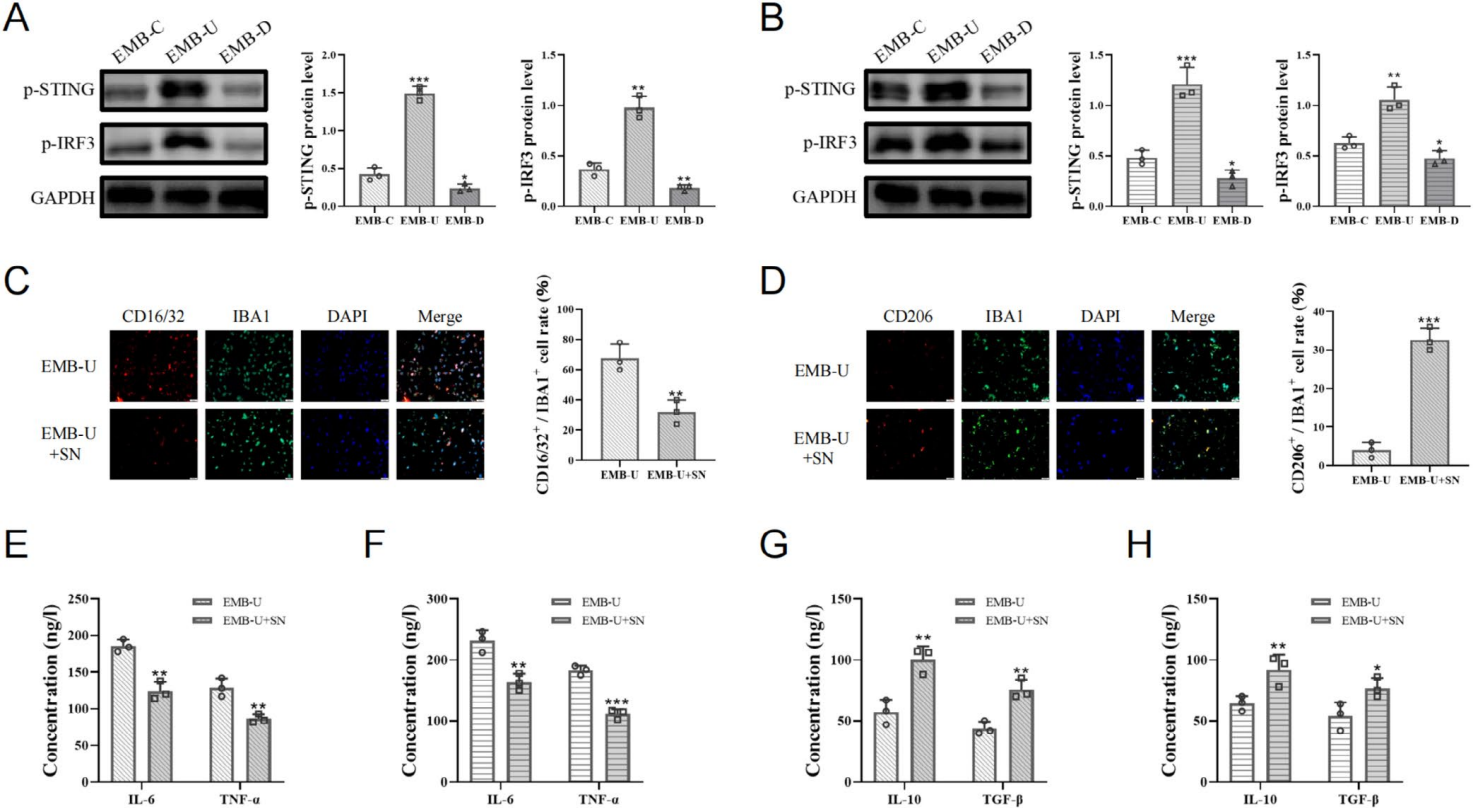
EMB counteracted the anti‐inflammatory effects of minocycline by activating the STING pathway. (A, B) The STING pathway activation levels in post‐OGD HMC3 cells (A) and BV2 cells (B) detected by Western blot analysis. (C, D) Immunofluorescence showing the proportion of double‐positive cells in post‐OGD EMB‐overexpressing HMC3 cells (C) and BV2 cells (D) treated with long‐term minocycline and the STING pathway inhibitor. Scale bar = 50 μm. (H) The levels of IL‐6, TNF‐α, IL‐10, and TGF‐β in post‐OGD EMB‐overexpressing HMC3 cells (E, G) and BV2 cells (F, H) treated with long‐term minocycline and the STING pathway inhibitor SN‐011. *p < 0.05; **p < 0.01; ***p < 0.001.

The STING pathway is implicated in microglial activation and inflammatory phenotype. We explored whether EMB regulates microglial polarization via the STING pathway. Western blot results showed that EMB modulates STING pathway activation in OGD‐treated microglia (Figure 5A,B). To assess the role of the STING pathway in EMB‐mediated minocycline resistance, we treated OGD‐treated EMB‐overexpressing cell models with long‐term minocycline combined with a STING pathway inhibitor. Immunofluorescence results indicated that STING pathway inhibition further downregulated CD16/32 in activated microglia and upregulated CD206, promoting the transition from inflammatory phenotype to anti‐inflammatory phenotype (Figure 5C–H). This suggests that the EMB/MCT4 axis counteracted the effects of minocycline by activating the STING pathway.

### 3.5 Long‐Term Minocycline Exerts Neuroprotective Effects on Neurons by Regulating Post‐Stroke Microglial EMB Levels

**FIGURE 6:**
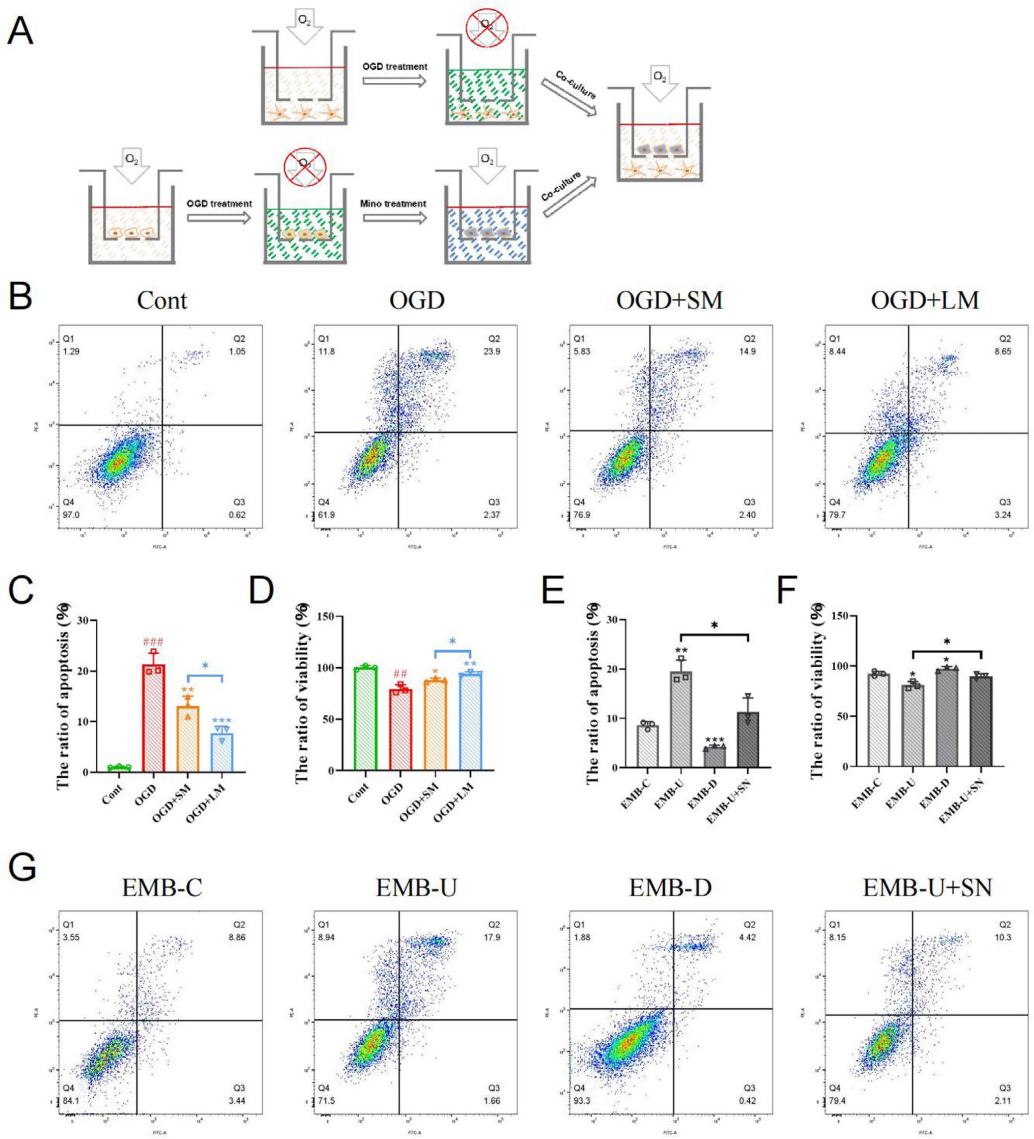
Long‐term minocycline‐regulated neuroinflammation‐induced neuronal injury through EMB. (A) Schematic diagram of cell co‐culture. (B–D) The apoptosis level (B, C) and viability (D) of SH‐SY5Y cells co‐cultured with HMC3 cells under different treatment conditions measured by flow cytometry and CCK8 assays. # indicates comparison between Cont and OGD groups, ##*p* < 0.01; ###*p* < 0.001. (E–G) The apoptosis level (E, G) and viability (F) of SH‐SY5Y cells co‐cultured with virus‐transfected HMC3 cells under OGD combined with long‐term minocycline treatment conditions measured by flow cytometry and CCK8 assays. *p < 0.05; **p < 0.01; ***p < 0.001.

To evaluate the effect on neurons influenced by poststroke microglial polarization induced by minocycline, we co‐cultured post‐OGD HMC3 cells exposed to minocycline with SH‐SY5Y cells (Figure 6A). The results showed that SH‐SY5Y cells co‐cultured with long‐term minocycline‐treated HMC3 cells had the lowest apoptosis level and highest viability compared to co‐cultures with untreated or short‐term treated HMC3 cells, indicating stronger neuroprotective effects of long‐term minocycline treatment (Figure 6B–D). In virus‐transfected cells, even after OGD and long‐term minocycline treatment, EMB‐overexpressing HMC3 cells still increased SH‐SY5Y cells apoptosis level and decreased cell viability, whereas co‐culture with EMB knockdown HMC3 cells promoted SH‐SY5Y cells survival. Meanwhile, inhibition of MCT4 partially alleviated the SH‐SY5Y cells injury caused by overexpression of EMB in HMC3 cells (Figure 6E–G).

### 3.6 Long‐Term Minocycline Treatment Promotes Post‐Stroke Recovery in Animal Models

**FIGURE 7:**
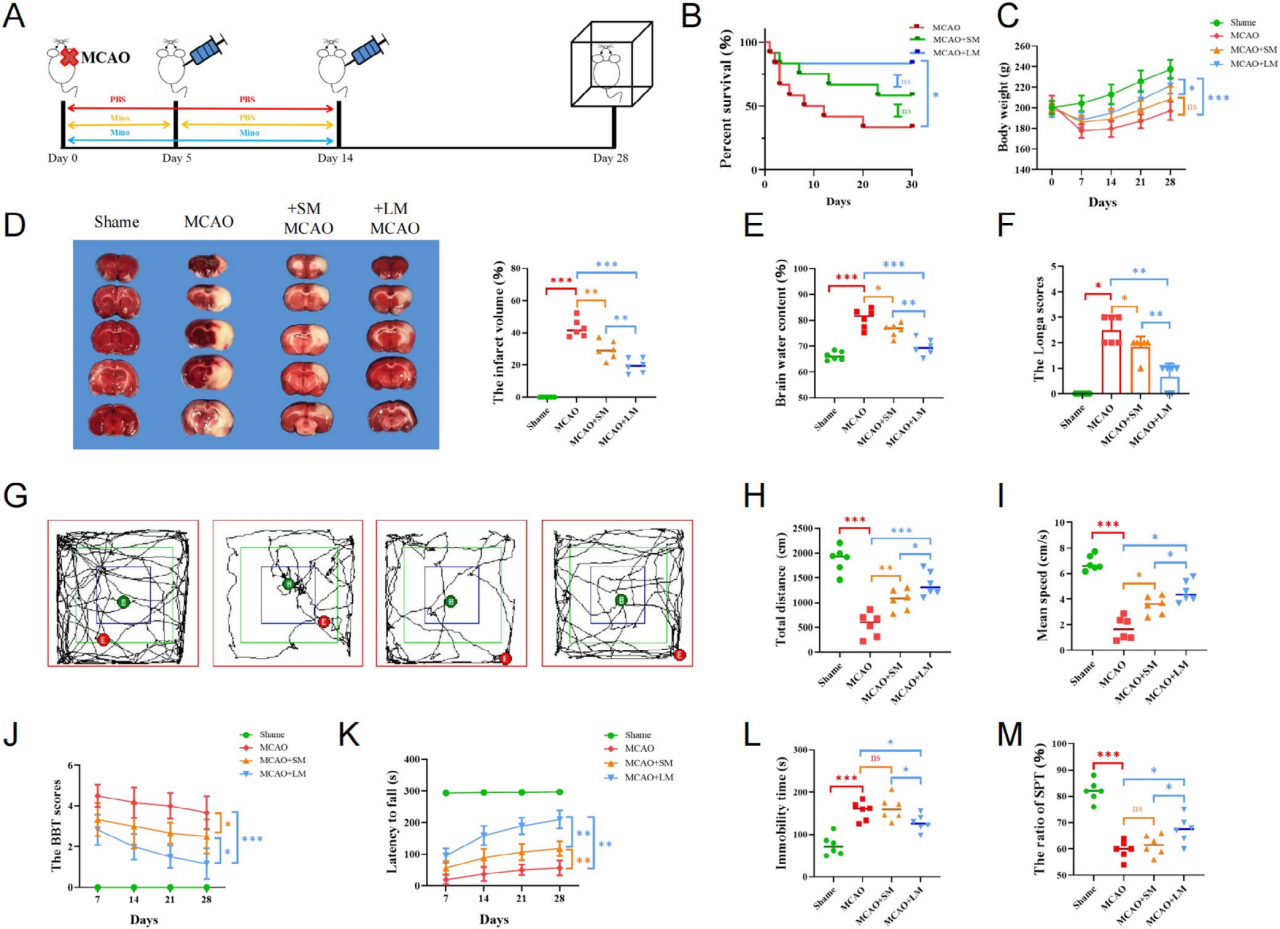
Long‐term minocycline treatment improved outcomes in animal models. (A) Schematic diagram of experimental intervention in animal models (*n* = 6 each group). (B) Survival curves of different animal model groups. (C) Body weight curves of different animal model groups. (D) Representative TTC‐stained images and infarct volume in different animal model groups. (E) Brain water content in different animal model groups. (F) Longa scores in different animal model groups. (G–I) Representative images, total distance, and average speed in open field tests of different animal model groups. (J) Balance beam test scores in different animal model groups. (K) Fall time in rotarod tests of different animal model groups. (L) Immobility time in forced swimming tests of different animal model groups. (M) Sucrose consumption in sucrose preference tests of different animal model groups. ns *p* > 0.05; *p < 0.05; **p < 0.01; ***p < 0.001.

Using a rat MCAO model, we administered minocycline intraperitoneally for 5 or 14 days and observed the outcomes at 28 days. Long‐term minocycline treatment significantly improved survival rates and body weight of model animals (Figure 7B,C). Infarct volume and brain water content were significantly lower in the long‐term treatment group compared to the control and short‐term treatment groups (Figure 7D,E). The Longa score was lower in the long‐term treatment group (Figure 7F). In the open field test, minocycline treatment increased the total distance traveled by animals, with long‐term treatment further increasing distance and average speed compared with short‐term treatment (Figure 7G–I). Rotating and balance beam tests also showed that extending minocycline treatment time promoted motor function recovery (Figure 7J,K). Additionally, long‐term minocycline improved performance in the forced swimming test and sucrose preference test, suggesting potential antidepressant effects, which were not observed in the short‐term treatment group (Figure 7 L,M).

**FIGURE 8:**
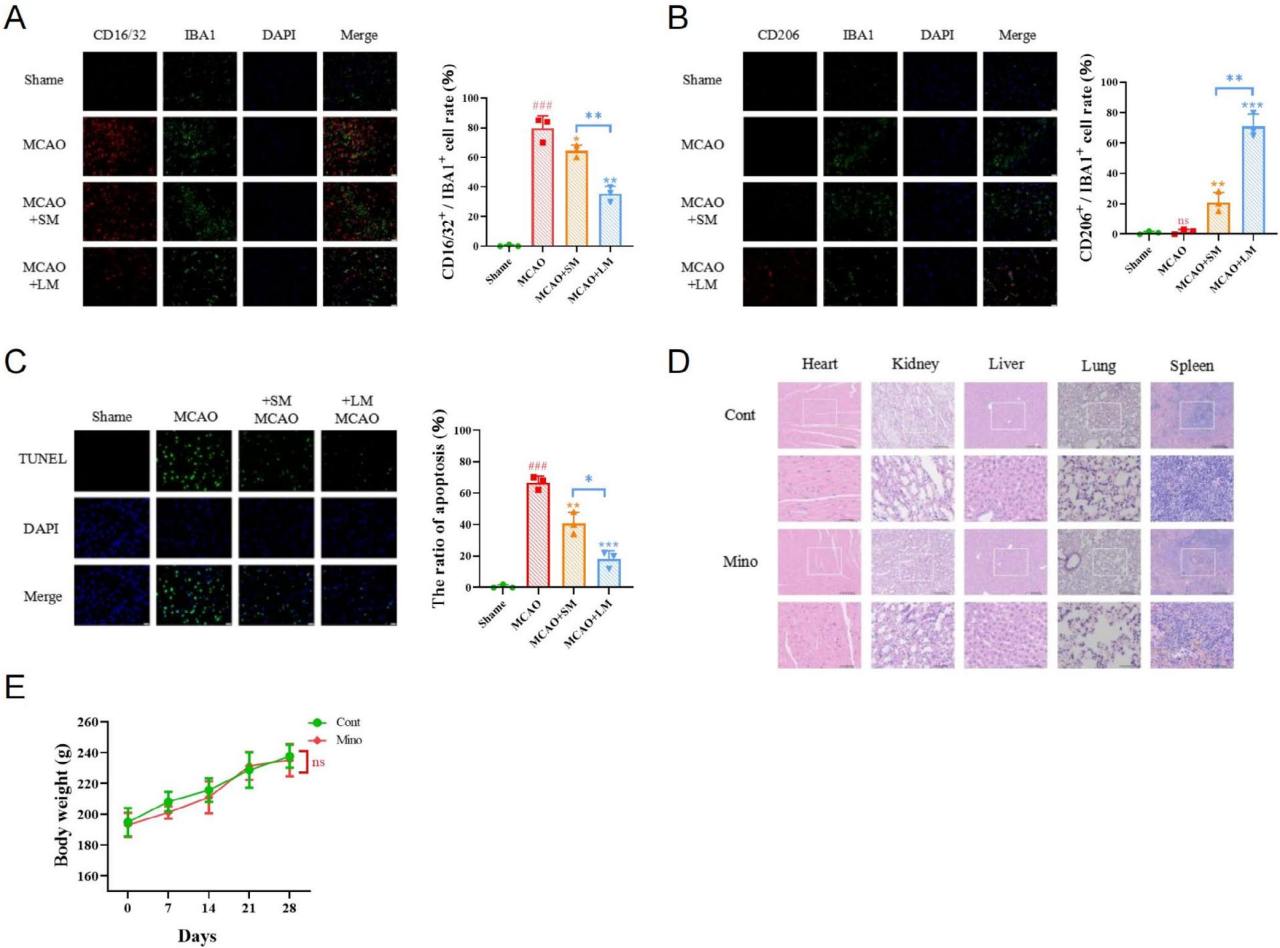
Neuroprotective effects and safety of long‐term minocycline treatment. (A, B) Immunofluorescence showing the proportion of double‐positive cells in the brains of different animal model groups. Scale bar = 20 μm. # indicates comparison between Sham and MCAO groups, ns *p* > 0.05; ###*p* < 0.001. (C) The apoptosis levels in the brains of different animal model groups detected by TUNEL assays. Scale bar = 20 μm. # indicates comparison between Sham and MCAO groups, ###*p* < 0.001. (D) HE staining results of major organs in different animal model groups. (E) Body weight curves of different animal model groups. ns *p* > 0.05; *p < 0.05; **p < 0.01; ***p < 0.001.

Immunofluorescence results showed that long‐term minocycline significantly inhibited the inflammatory phenotype, promoted the anti‐inflammatory phenotype, and reduced apoptosis in the brains of model animals, whereas short‐term treatment was less effective (Figure 8A–C), but it did not significantly affect major organs or body weight, indicating its biosafety (Figure 8D,E).

## 4 Discussion

Recent studies have shown that minocycline can inhibit microglial activation [10, 57]. Given the role of excessive microglial activation in stroke‐induced neuronal damage, numerous clinical trials have investigated the efficacy of minocycline as a therapeutic agent in stroke. However, most current studies use a minocycline treatment duration of 3–5 days [19, 20, 21]. Since microglia remain activated for an extended period following a stroke [18, 19], we hypothesized that a longer treatment duration could enhance minocycline's therapeutic effect. To test this, we chose the standard 5‐day clinical treatment period as the short‐term treatment duration [19, 20, 21]. Considering that microglial activation remains significant after 2 weeks of cerebral ischemia, we selected 14 days for the long‐term treatment duration [17, 18]. Based on CCK8 assay results and to minimize the impact of cell passage and other procedures, we chose 6 h and 18 h for cell treatment times, respectively. Our study demonstrated that extending the minocycline treatment duration significantly improved neuroprotective effects in both cell and animal models, as evidenced by inhibiting the inflammatory phenotype of microglia, promoting the anti‐inflammatory phenotype, and reducing proinflammatory factors, ultimately leading to reduced neuronal damage. These findings suggest that the anti‐inflammatory effects of minocycline are time‐dependent and that stroke injury induces prolonged microglial activation.

In their resting state, microglia primarily generate energy through the relatively slow, but efficient oxidative phosphorylation pathway. Upon activation, microglia rapidly shift their energy acquisition to glycolysis [18, 19]. The upregulated glycolysis promotes the transformation of activated microglia to the inflammatory phenotype, making the inhibition of microglial glycolysis a promising therapeutic target [31, 58, 59]. However, research on microglial glycolysis and metabolic reprogramming in stroke remains limited. Glycolytic reprogramming under hypoxic conditions enhances microglial activation and exacerbates post‐ischemic brain inflammation through various factors such as hexokinase 2, phosphoglycerate kinase 1, serglycin, and chemokine‐like factor 1 [29, 31, 60, 61]. Shifting microglial metabolism toward oxidative phosphorylation has been shown to promote brain function recovery after stroke [61]. It is noteworthy that similar effects on brain injury are observed in subarachnoid hemorrhage, where the aerobic glycolysis‐dependent inflammatory phenotype of microglia is significantly increased [59], highlighting the importance of microglial glycolytic metabolism in neuroinflammatory injury. Our study confirmed that stroke induces EMB expression in microglia, and elevated EMB levels further enhance glycolytic metabolism in microglia, thereby worsening inflammation. EMB is reported to affect cellular glycolytic metabolism mainly through the MCT family [36, 62]. The MCT family, crucial for lactate transport, plays a significant role in regulating glycolytic metabolism in both normal and diseased cells [63, 64]. Microglia exhibit abundant MCT expression, such as MCT1 and MCT4, suggesting that the MCT family might be key in EMB‐mediated microglial glycolytic metabolism. Previous studies have only investigated the relationship between MCT1 and microglial activation in stroke, finding that MCT1 correlates positively with the inflammatory phenotype marker INOS in microglia in stroke models [65]. Recent studies indicate that MCT4 is an essential lactate transporter in microglia, and the absence of MCT4 disrupts lactate metabolism within cells, affecting neuronal synaptic modification and potentially causing epilepsy or anxiety [37]. Based on this, we hypothesized that EMB in microglia might influence glycolytic metabolism by regulating MCT4. Our results confirmed that, like MCT2, MCT4 is also critical in EMB‐mediated glycolytic metabolism and neuroinflammation, and inhibiting MCT4 significantly reduces the inflammatory phenotype in microglia.

We further investigated how EMB/MCT4‐induced glycolytic metabolism influences microglial activation. The STING pathway appears to play a pivotal role. Recent studies indicate that excessive activation of the STING pathway can trigger type I interferon responses, leading to harmful inflammation. Inhibiting the STING pathway in poststroke microglia can facilitate the shift from the inflammatory phenotype to the anti‐inflammatory phenotype, thereby mitigating neuroinflammatory damage [66, 67, 68]. Activation of the STING pathway is often associated with abnormal increases in cellular glycolytic levels, though the specific regulatory mechanism remains unclear [43, 44, 69]. In this study, we found that EMB upregulates the STING pathway in OGD‐treated cell models, and using a STING pathway inhibitor further reduced the inflammatory response in microglia induced by EMB overexpression. This suggests that the EMB/MCT4 axis mediates the microglial inflammatory phenotype in stroke through the STING pathway.

## 5 Conclusion

In conclusion, extending the treatment duration of minocycline appropriately can more effectively inhibit neuroinflammatory injury caused by microglial activation in stroke. In the process, glycolysis levels were downregulated through the EMB/MCT4/STING axis, thereby suppressing neuroinflammatory responses induced by microglia.

## Author Contributions

The conception and design of the work were prepared by Dunjing Wang and Jing Liu. The experiment was performed by Bo Cheng, Shangqi Liu, Ling Gao, Ziwen Zhu, Yang Yang, and Rui Ma, while the analysis and interpretation of data were done by Ning Xin, Zhenying Shang, and Zixiang Xu. Drafting of the manuscript was done by Dunjing Wang and Bo Cheng. All the authors revised the manuscript and approved the submission.

## Ethics Statement

All animal experiments were approved by the Experimental Animal Ethics Committee of Xuzhou Medical University (202306 T001) and conducted in accordance with relevant ethical standards and guidelines.

## Consent

Human Protein Atlas and GEO belong to public databases. The patients involved in the database have obtained ethical approval. Users can download relevant data for free for research and publish relevant articles. Our study is based on open‐source data, so there are no ethical issues or other conflicts of interest.

## Conflicts of Interest

The authors declare no conflicts of interest.

## Supporting information


**Figure S1.** EMB is highly expressed in microglia and is associated with the severity of stroke.

## Data Availability

All data generated or analyzed during this study are included in this published article.
