# Supplementary material for: Long‐Term Minocycline Treatment Exhibits Enhanced Therapeutic Effects on Ischemic Stroke by Suppressing Inflammatory Phenotype of Microglia Through the EMB/MCT4/STING Pathway
Source: CNS Neurosci Ther. 2025 Mar 26;31(3):e70328. doi: 10.1111/cns.70328 (PMC11937927; doi:10.1111/cns.70328)
Supplement: Supplementary file 1 — Figure S1. EMB is highly expressed in microglia and is associated with the severity of stroke. [file CNS-31-e70328-s001.docx]

**
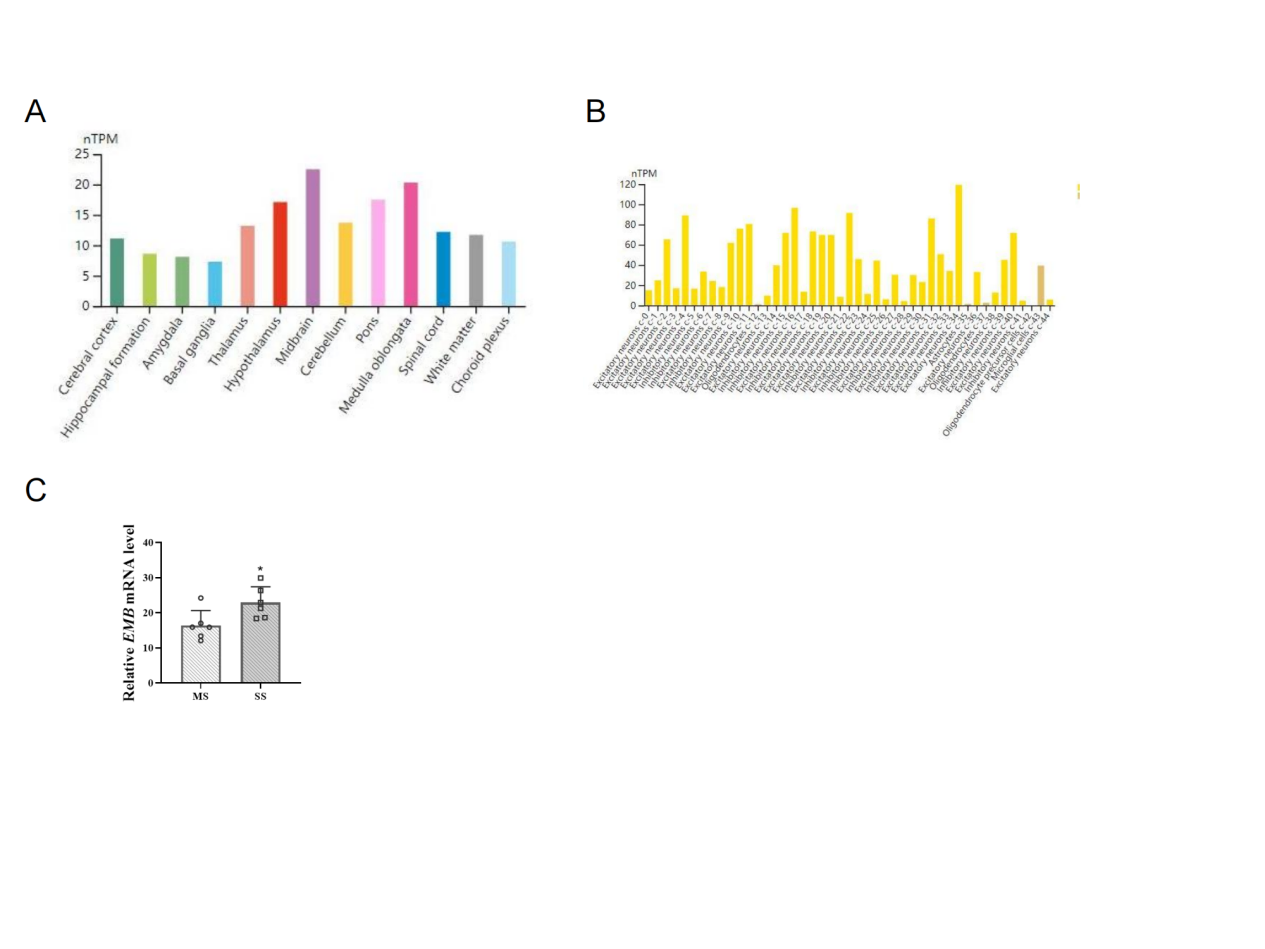
Figure S1. EMB is highly expressed in microglia and is associated with the severity of stroke.**

A, B: EMB mRNA levels in various brain regions (A) and cell types (B) in the human brain [70,71]. Image credit: Human Protein Atlas. Image available from v23.0.proteinatlas.org. C: EMB mRNA levels in patients with moderate and severe stroke [56]. Data sourced from the GEO database, Series GSE202518.

* p < 0.05.
